# Last Stop Before Exit – Hepatitis C Assembly and Release as Antiviral Drug Targets

**DOI:** 10.3390/v2081782

**Published:** 2010-08-24

**Authors:** Birke Andrea Tews, Costin-Ioan Popescu, Jean Dubuisson

**Affiliations:** 1 Hepatitis C Laboratory, Center of Infection and Immunity of Lille, University Lille Nord de France, CNRS UMR8204, INSERM U1019, Pasteur Institute of Lille, 1, rue du professeur Calmette, BP447, 59021 Lille, France; E-Mails: popescu80@hotmail.com (C.-I.P.); jean.dubuisson@ibl.fr (J.D.); 2 Institute of Biochemistry of the Romanian Academy, Splaiul Independentei 296, 060031, Bucharest, Romania

**Keywords:** Hepatitis C, assembly, release, antivirals

## Abstract

Chronic Hepatitis C infection is a global health problem. While primary infection is often inapparent, it becomes chronic in most cases. Chronic infection with Hepatitis C virus (HCV) frequently leads to liver cirrhosis or liver cancer. Consequently, HCV infection is one of the leading causes for liver transplantation in industrialized countries. Current treatment is not HCV specific and is only effective in about half of the infected patients. This situation underlines the need for new antivirals against HCV. To develop new and more efficient drugs, it is essential to specifically target the different steps of the viral life cycle. Of those steps, the targeting of HCV assembly has the potential to abolish virus production. This review summarizes the advances in our understanding of HCV particle assembly and the identification of new antiviral targets of potential interest in this late step of the HCV life cycle.

## 1. Introduction

HCV is the agent that causes Hepatitis C, a disease that affects around 130 million people worldwide. Primary infection with HCV often shows only mild symptoms, but in the majority of patients, the infection becomes chronic and leads to liver cirrhosis, which often results in liver cancer [1]. HCV infection is currently one of the major reasons for liver transplantation in Europe and in the United States. There is no vaccine available to protect against HCV and vaccine development is severely hampered by the weak immune response to HCV infection, by the wide variety of HCV genotypes, and by the lack of an immunocompetent small animal model for HCV. HCV infection is currently treated with pegylated interferon α and ribavirin, but treatment is only effective in about half of the infected patients, and the success of the therapy is highly dependent on the genotype of HCV [1]. Furthermore, current treatment, which is mainly immunostimulatory, targets cellular processes rather than viral proteins, and treatment is therefore often accompanied by several, sometimes severe, side effects. Obviously, there is an urgent need for the development of new antivirals that will achieve better treatment efficacy. In particular, antiviral drugs that target viral proteins and viral specific processes, instead of broad host processes, are required.

**Figure 1.**
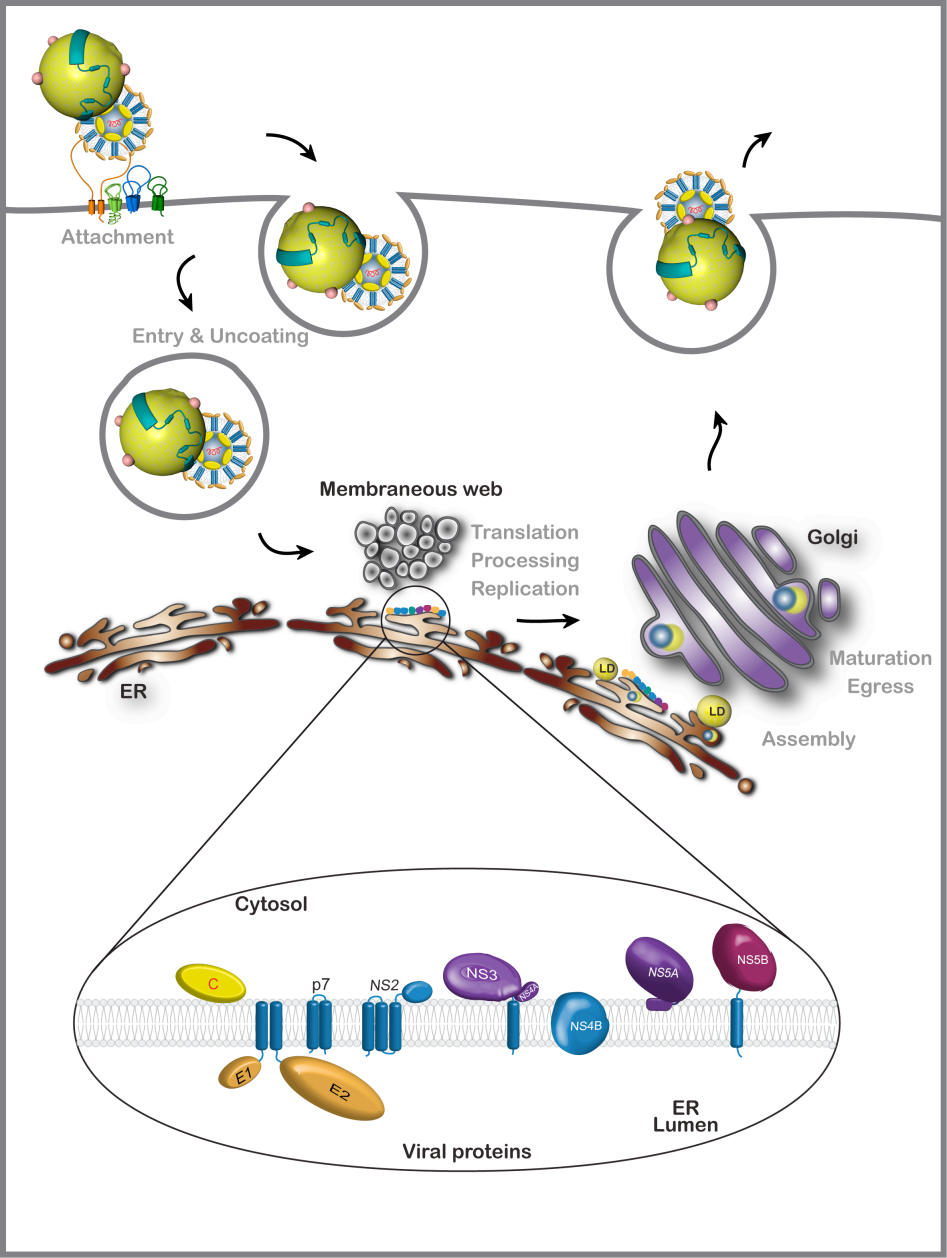
Schematic representation of the viral life cycle and viral proteins. HCV is closely associated with very light density lipoprotein (VLDL) particles. Entry of this lipoviroparticle needs at least four essential entry factors, Scavenger Receptor Class B Type I, CD81, Claudin, and Occludin. Receptor binding is followed by clathrin-mediated endocytosis. Viral RNA is released into the cytosol and serves as a template for the production of the viral proteins (see inset) and for the negative strand, which will serve to produce new viral genomic RNA (in close proximity with ER-derived membranes). Assembly starts with core and NS5A recruitment to lipid droplets (LD), followed by particle formation. The virion interacts with VLDL particles. Lipoviroparticles undergo maturation during the transport through the Golgi apparatus and become more lipidated.

HCV is a small enveloped RNA virus belonging to the *Flaviviridae* family, more precisely to the genus Hepacivirus [2]. HCV has a genome of 9600 nucleotides, which encodes a single polyprotein that is co- and post-translationally cleaved to produce different viral proteins. The N-terminal part of the polyprotein contains the structural proteins, the core protein (C), and the envelope proteins (E1 and E2). The rest of the polyprotein contains the p7 polypeptide, followed by the non-structural proteins NS2, NS3, NS4A, NS4B, NS5A, and NS5B (see Figure 1). In addition, infected patients produce antibodies against an alternate reading frame protein starting from the core encoding region [3]. So far, no function has been attributed to this protein and its identification remains elusive. The viral polymerase NS5B is error-prone, leading to a wide variability amongst different HCV genomes. Based on those sequence differences, HCV can be grouped into seven genotypes [4].

HCV infection of the hepatocyte begins with a complex interaction of the virion with a series of cellular entry factors (for a review see [5]). The viral particle is then internalized by clathrin-mediated endocytosis [6,7]. This is followed by the release of the viral RNA into the cytosol. The genome is then translated and processed to generate the viral proteins. The non-structural proteins assemble the replication complex, which is tightly linked to endoplasmic reticulum (ER)-derived membranes [8–10]. With the progressive accumulation of new genomic RNA and structural proteins, progeny viral particles are formed in an intracellular compartment and they are released from the cell through the secretory pathway. The viral lifecycle can be divided in three major phases: (i) entry and uncoating of the virus, (ii) translation and replication of the viral genome, and (iii) assembly and egress of the new viral particles (Figure 1). All of these steps are possible targets for antiviral drugs, which could greatly enhance current therapies.

## 2. HCV Assembly

### 2.1. Viral components of the assembly machinery

As components of the HCV particle, the RNA genome, the core protein, and the envelope glycoproteins are essential elements in the assembly process. Since non-structural proteins NS3 to NS5B have essential roles in replication, it was long thought that these proteins were solely responsible for catalyzing the accumulation of genomic RNA molecules that could subsequently be packaged by the structural proteins. This hypothesis was initially supported by the ability of subgenomic replicons lacking the structural region to efficiently undergo RNA synthesis [11,12]. However, whereas replication is independent of the structural proteins, the packaging of HCV genomes into infectious particles seems to require more than just the physical components of the virion. Indeed components of the replication complex, as well as the non-structural proteins p7 and NS2, are involved in HCV morphogenesis. Interestingly, this dual function of the non-structural proteins is emerging as a general feature of the assembly process in the *Flaviviridae* family [13].

### 2.2. The viral particle factory

For a long time, it has been known that the HCV core accumulates around lipid droplets (LDs), which are storage organelles for neutral lipids, such as triglycerides and cholesterol esters [14–16]. However, it is only recently that LDs were identified as a central element of the viral particle factory [17]. The cleavage between the core protein and E1 in the polyprotein is mediated by a signal peptidase. However, the core protein needs to be further cleaved by a signal peptide peptidase in order to interact with LDs. This process leads to the mature core protein [18–20]. Based on amino acid distribution and hydrophobicity plots, the mature form of the HCV core protein can be divided into two domains, D1 and D2 [21–23]. The D1 domain is rich in basic residues and is comprised of the N-terminal two-thirds of the core, whereas D2 encompasses the C-terminus and is more hydrophobic. The basic clusters of the D1 domain are thought to enable core-RNA interaction [18,24,25]. Furthermore, core-core interaction has been mapped to amino acids in the middle of the core sequence [26], whereas the D2 domain contains two amphipathic helices and a hydrophobic loop that are necessary for interaction with LDs [22,27]. Interaction of the core protein with LDs is now recognized as essential for infectious virus production [27,28]. However, an inverse correlation has been observed between the extent of core-LD association and virus production, suggesting that these interactions are dynamic and regulated during virus assembly [29]. Furthermore, core proteins may also contribute to HCV assembly by inducing the relocation of LDs to the periphery of the nucleus and of those in close contact with the replication complex and the ER membranes [30].

In addition to the core protein, some viral non-structural proteins like NS5A and NS3 are also found around LDs in HCV infected cells [17]. However, the core-LD association is essential for the recruitment of these other viral proteins and for virus production. The core protein has indeed been shown to recruit the non-structural proteins around the LDs by interacting with NS5A [31–33]. NS5A might therefore be the scaffold for the recruitment of the other non-structural proteins to the LDs. This process can explain how replication complexes, which contain the newly synthesized RNA genome and the non-structural proteins, are brought into contact with the core. Importantly, NS5A is emerging as a protein that plays a double role in both the replication and the assembly processes. NS5A is a membrane-associated RNA-binding phosphoprotein composed of three domains and an N-terminal membrane anchor [34–38]. Within NS5A, domain I has been proposed to be involved in RNA replication, whereas domain III (DIII), which is dispensable for RNA replication, is a central player in HCV assembly [31–33]. It is increasingly clear that mutations that stabilize replication events tend to inhibit virion production. This indicates a close relationship between these processes [39–41]. A model has been proposed in which NS5A is maintained in the functional replicase via association with host factors, but dissociates upon phosphorylation, leading to viral particle assembly [42,43]. In fact, phosphorylation of NS5A at a cluster of serine residues in DIII is important for the interaction of NS5A with the core [44,45] and thus is critical for the start of the assembly process.

Other proteins in the replication complex are also involved in HCV assembly. The identification of adaptive mutations in NS3 suggested a role for this protein in the assembly process [46–48]. NS3 is a well characterized viral protein that has protease and helicase activities. NS3 cleaves the polyprotein between the different non-structural proteins, starting with NS3, and is thus the prime target for antiviral drugs that aim to abolish polyprotein processing.

Current models propose that viral genomes are transported as part of the replication complexes to the core/NS5A-containing LDs, and that assembly takes place there. Replication occurs in dedicated ER-derived vesicles [8–10]. HCV infection induces clustering of LDs around the nucleus and in close contact with the ER, which might facilitate assembly by bringing replication complexes and core-coated LDs into close contact. Currently, we lack information regarding core-RNA association, formation of the nucleocapsid, and the mechanism by which core-RNA particles are packaged in the lipid envelope containing the envelope glycoproteins. Models propose that the interaction of core and genomic RNA via replication complexes at the LDs leads to the nucleocapsid formation with the help of NS5A close to the LDs. These particles then bud into the ER lumen, where they obtain their lipid envelope, which contains the viral glycoproteins.

### 2.3. Involvement of p7 and NS2 in HCV assembly

Besides the structural proteins and the viral components of the replication complex, the remaining HCV proteins, p7 and NS2, are also essential for HCV morphogenesis. Since NS2 is dispensable for HCV RNA replication, and it does not seem to be incorporated into viral particles [12], the protein has been suspected to be involved in the assembly process of the HCV particle. NS2 is a small membrane-bound protein with a C-terminal cytosolic domain that is bound to an N-terminal transmembrane domain that might contain three transmembrane segments [49]. For a long time, the only known function of NS2 was its role in polyprotein processing as part of the auto-protease that cleaves *in cis* between NS2 and NS3. However, data from several studies that have used the HCV cell culture (HCVcc) system suggest the specific involvement of NS2 during the assembly of infectious HCV particles [49–53]. Indeed, compensatory mutations accumulating within the N-terminal domain of NS2 following transfection of an inter-genotypic, 1a/2a chimeric viral RNA have been shown to enhance the specific infectivity of secreted virus particles [46]. Furthermore, the deletion of the NS2 sequence was shown to block infectious virus production by an otherwise viable bi-cistronic HCV RNA that no longer required the NS2-NS3 auto-protease for genome amplification [51]. Structural and functional characterization of the NS2 transmembrane domain has shown that this domain is essential for infectious virus production [49]. In addition, the NS2 protease domain, but not its catalytic activity, is also essential for infectious virus assembly [49,51]. A reverse genetic analysis of NS2 in the HCVcc system also indicated that the NS2 protein contributes to virus particle assembly via opposing interactions with the E1E2 glycoprotein and the NS3-NS4A enzyme complexes [48]. Finally, it has been proposed that NS2 is involved in a late post-assembly maturation step [53]. Although these studies strongly support a role for NS2 during virus assembly via a complex network of interactions involving other viral structural and non-structural proteins, the details of that role and how NS2 might act during the production of infectious virus particles remain unknown.

Another viral protein involved in a late step of the HCV life cycle is the p7 polypeptide. HCV p7 is comprised of two helical domains connected by a polar loop [54]. The proteins assemble as a hexamer [55]. HCV p7 is dispensable for genomic replication and there is no clear evidence of its incorporation into the viral particle. p7 has been shown to form cation-selective channels in artificial membranes [56–58], a property that likely depends on the oligomerization of the protein [55,59,60]. A detailed structure of an HCV p7 ion channel has recently been obtained by electron microscopy [55]. The density map at a resolution of around 16 Å reveals a flower-shaped protein architecture with protruding petals oriented toward the ER lumen. This broadest part of the channel presents a comparatively large surface area, which can provide interaction sites for cellular proteins, viral proteins, or both. Mutation analyses of p7 in the HCVcc system revealed a role for this polypeptide in virus assembly and release [51,52,61]. However, as with NS2, the precise role of p7 in HCV morphogenesis remains undetermined.

### 2.4. HCV envelope glycoproteins and virion assembly

HCV envelope glycoproteins, E1 and E2, are major components of the viral particle. These proteins are sufficient to confer infectivity to HCV pseudotyped retroviral particles [62,63], and they are essential for HCV infectivity in cell culture [64]. Each protein consists of a large N-terminal ectodomain and a C-terminal transmembrane domain that anchors each glycoprotein in a lipid bilayer. Both ectodomains are heavily glycosylated [65] and their structure is stabilized by disulfide bridges [66]. Both transmembrane domains contain ER retention signals [67,68] that are responsible for their subcellular localization [69]. From the start, the fates of E1 and E2 glycoproteins are closely linked, as both proteins are expressed consecutively in the viral polyprotein [70]. Characterization of these glycoproteins using heterologous expression systems suggested that the E1E2 non-covalent heterodimer is the functional complex for HCV entry [71,72]. However, in the context of the HCVcc system, virion-associated E1 and E2 envelope glycoproteins form large covalent complexes stabilized by disulfide bridges, whereas the intracellular forms of these proteins assemble as non-covalent heterodimers [73]. The presence of disulfide bridges between HCV envelope glycoproteins suggests that lateral protein-protein interactions, assisted by disulfide-bond formation, might play an active role in the budding process of the HCV particle. Interestingly, the functional subviral HCV particles can be produced only when the HCV envelope glycoproteins are expressed in lipoprotein-producing cell lines [74,75], supporting the idea that the HCV envelope glycoproteins play an active role during the budding process.

### 2.5. Very low density lipoproteins (VLDL) and HCV assembly

The secreted viral particles that are found in cell culture supernatant or in the blood of infected patients are rich in triglycerides and contain apolipoprotein B (ApoB) and apolipoprotein E (ApoE), in addition to the viral structural components [76,77]. These particles are called lipoviroparticles and are the product of a close association between VLDL assembly and HCV assembly. Importantly, HCV infectivity correlates with the extent of the viral particle lipidation. Indeed, particles recovered from animals infected with HCVcc virus present lower density and increased specific infectivity compared to the original inoculum [78].

Lipoproteins are the form in which hydrophobic lipids can be transported in the bloodstream. VLDL are produced in the liver to export cholesterol and triglycerides. VLDL production starts with the expression of ApoB100, a highly hydrophobic protein, which is lipidated by the microsomal triglyceride transfer protein (MTP), leading to the formation of a poorly lipidated initial form of VLDL. These pre-VLDL are thought to fuse with luminal triglyceride droplets in an ApoE dependent manner, before being secreted from liver cells [79].

ApoB, ApoE, and MTP have been found in close association with replication complexes [77,80]. Their importance for HCV assembly has been verified by siRNA experiments and inhibitor studies. Several initial reports showed that the inhibition of both ApoB secretion and MTP activity inhibit HCV secretion [80–82], whereas later reports showed a direct correlation between ApoE and HCV secretion and direct interaction of NS5A and ApoE without any effect of ApoB on viral production [83,84]. ApoB and ApoE secretion in VLDL assembly are linked processes and influence each other, which might explain the initially detected effect of ApoB silencing (especially as ApoB is involved in the initial assembly of VLDL, and ApoE intervenes at a later step). Furthermore, the differences might in part be due to the use of slightly different viruses, as the virus used to show the ApoB independence was a highly replicating cell culture adapted virus. Nevertheless, further investigations into the role of the proteins in HCV assembly and release, as well as into VLDL assembly, are necessary to elucidate the exact mechanisms for these processes.

### 2.6. Viral particle transport and release

After budding, HCV particles are transported through the cellular secretory pathway to the extracellular milieu. The secretion and maturation of the virions have been well described for the flaviviruses [85]. However, the maturation of HCV particles seems to be fundamentally different from that of flaviviruses. Intracellular infectious virus can be detected in HCV-infected cells [86], suggesting that these virions mature immediately, or rapidly, after their formation. Furthermore, in contrast to flaviviruses, post-assembly cleavage events in one of the envelope proteins do not seem to be involved in HCV maturation. Finally, despite undergoing low-pH-dependent entry [6], HCV particles are acid resistant, which indicates that unknown factor(s) during uptake, rather than during egress, prime the envelope proteins for fusion [87]. Importantly, even if HCV virions are infectious soon after they are formed, they undergo physical modification during egress, as shown by their decrease in density as they pass through the secretory pathway [86]. Due to their association with VLDL during the assembly process, it is believed that these changes in virion density are due to the extensive lipidation of the VLDL moiety in the secretory pathway.

## 3. Drug targets

Although the cloning of the HCV genome more than 20 years ago [88] allowed a rapid analysis of its genomic organization, as well as a biochemical characterization of its proteins [89], the lack of a cell culture system to efficiently amplify this virus has long been a major obstacle for the study of the HCV life cycle. Early research into the viral life cycle used surrogate systems, e.g., the related pestivirus bovine viral diarrhea virus (BVDV) [90]. However, several tools have been progressively developed to study this virus. The first major advance in the field was the development of a subgenomic replicon corresponding to a replicative mini-genome, which was the first tool available to study genomic replication [12]. This discovery was followed by the development of retroviral particles pseudotyped with the HCV envelope glycoproteins (HCVpp) to study HCV entry [63,91,92]. More recently, a cell culture system that allows for a relatively efficient amplification of HCV (HCVcc) has finally been developed [64,93,94]. This system is based on the transfection of the human hepatoma cell line Huh-7 with genomic HCV RNA, which was derived from a viral genome cloned from HCV. This HCV was isolated from a Japanese patient with fulminant hepatitis (JFH-1 isolate). Data accumulated with the HCVcc system sheds light on the life cycle of this virus. However, some limits still remain. So far, no other cell culture infectious virus has been isolated, and chimeric viruses seem to need the JFH-1 non-structural proteins to be infectious in cell culture. Additionally, only a limited number of cell lines are susceptible to HCVcc infection. Furthermore, there are distinct differences in the lipidation and infectivity profile of infectious viruses derived from cell culture when compared to those derived from infected patients/animals [78]. The only animal naturally susceptible to HCV is the chimpanzee, but a chimeric mouse model based on uPa/SCID mice colonized with human liver cells has been developed [95, 96]. Nevertheless, HCVcc allows for the successful analysis of all steps in the viral life cycle and has become a very useful tool in the screening of antiviral drugs.

The current therapy against HCV results in a Sustained Virologic Response (SVR) for a fraction of the treated patients. This response is strongly dependent on the genotype of the virus. Thus, the SVR for genotype 1, which is the most frequent genotype in the Western world, is around 50% [1]. Specifically targeted therapy against Hepatitis C (STAT-C) targets multiple steps and is quite advanced in the drug pipeline. This multiple targeting strategy is similar to the Highly Active Antiretroviral Therapy strategy (HAART) that is used for treating the human immunodeficiency virus (HIV). Drug development closely mirrors the development of HCV research tools. The biochemical and structural characterization of the NS3/4A protease and the NS5B polymerase, in the context of the replicon system, led to the development of several promising new antiviral molecules that block HCV replication [97], and which are most advanced in clinical studies. The accumulation of knowledge helped by the use of the HCVpp system is also leading to the preclinical development of molecules blocking viral entry [98]. Research into assembly and release only became possible with the first cell culture infectious virus in 2005 [64,93,94], and is therefore lagging. Nevertheless, interfering with HCV assembly also holds promise for drug design and discovery. The possibility of interference is becoming more accessible as the understanding of the molecular mechanisms underlying HCV morphogenesis advances.

Although several protease and polymerase inhibitors are currently in advanced clinical studies [99], the need for additional anti-HCV drugs remains. The emergence of strains resistant to these new drugs is fast. Therefore, it is important to increase the pool of potential antivirals directed against HCV.

Drugs targeting viral assembly can have several advantages. The prevention of assembly might lead to a rapid drop in viral titers in the blood of infected patients after the start of the treatment because components that were already produced can no longer be packaged into infectious particles. This, like other antivirals, will limit viral spreading, but can work more quickly than replication inhibitors. Furthermore, infected cells treated with assembly inhibitors might induce a stronger antibody based immune response than those of untreated cells. This is because an excess of accumulated and then degraded viral protein in the cell leads to higher antigen presentation. Similarly, the innate immune response, which is curiously weak in HCV, might be activated due to an excess of viral RNA in infected cells. This, however, can be counteracted by viral proteins that inhibit the Interferon β response (e.g., the cleavage of MAVS by NS3/4A [100]). However, the intracellular accumulation of viral genomes and proteins might also prove to be a problem for assembly inhibitors, as high concentration might favor unorthodox assembly and escape.

More important than the advantages or disadvantages of single therapy is the consideration of a combined therapy that targets different steps of the viral life cycle. This can limit the emergence of resistance mutation, as selection pressure is applied not to a single viral function, but to several vital processes. For example, in combination therapy, even genomes that replicate in spite of polymerase inhibitors would not be packaged without the accumulation of further mutations to overcome assembly inhibition. Combined with the current treatment, which is mostly immunostimulatory, this might even lead to the complete elimination of the virus. Combination therapy with three different drugs is currently the standard for treatment against HIV (WHO guidelines). HIV is another virus for which the assembly step is slowly emerging as a possible target for drugs. Indeed, several betulinic acid derivatives, which inhibit virion formation by inhibiting gag cleavage, made it into clinical trials [101]. However, for most pathogens, the development of assembly inhibiting drugs is generally lagging.

The assembly process contains several attractive targets for drug development. This ranges from the obvious viral proteins, which are essential for viral assembly and release, to the cellular factors that are important for these processes. The major proteins of assembly and the drugs directed against them are outlined in the following section.

### 3.1. Possible viral targets and existing drugs

**Figure 2.**
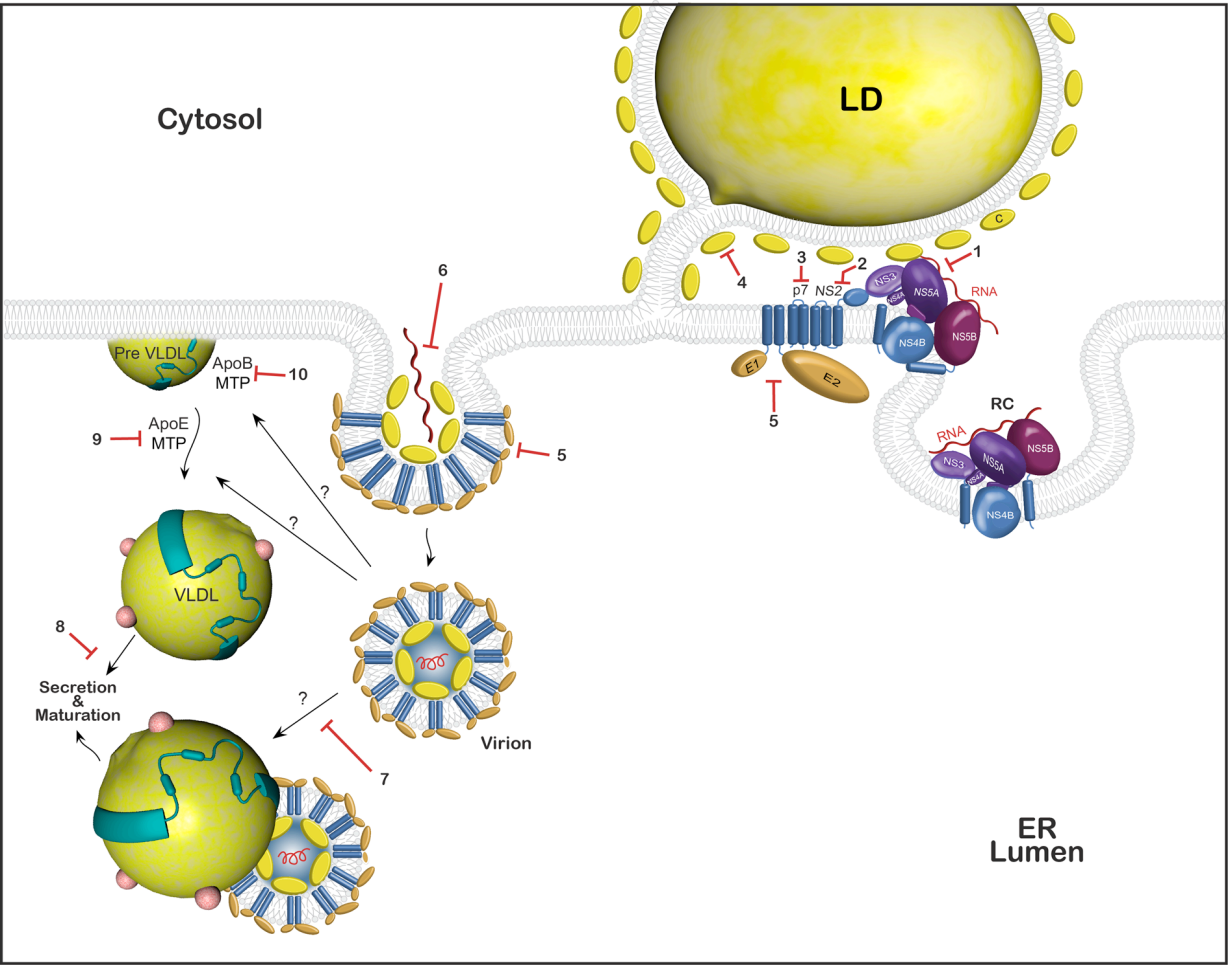
Schematic representation of the assembly and release of HCV. Replication occurs at dedicated ER-derived membranes. Core and NS5A interact with lipid droplets and recruit the other non-structural proteins, and with them, the replication complex. Core and genomic RNA interact and oligomerize, and must interact with the envelope glycoproteins to form the viral particle. The viral particle interacts with the VLDL. VLDL assembly is depicted on the left side. ApoB interacts with triglycerides in an MTP-dependent manner to form a pre-VLDL, which will accumulate more lipids and ApoE in an MTP dependent process. Both the VLDL and the virus continue to undergo maturation during their passage through the secretory pathway. Red blocks mark potential drug targets in this process: (1) inhibition of the interaction of NS5A with LD or the other non-structural proteins, thus blocking their recruitment to LDs; (2) inhibition of NS2, a late stage block of assembly; (3) inhibition of p7 (BIT225 for example); (4) inhibition of the interaction of the core with LDs; (5) inhibition of the maturation of envelope proteins (inhibitors of α-glycosidases); (6) inhibition of core oligomerization and interaction with RNA; (7) inhibition of virus-VLDL association; (8) inhibition of VLDL secretion; (9) inhibition of VLDL formation from pre-VLDL targeting ApoE and MTP; (10) inhibition of the formation of pre-VLDL by the MTP dependent association of triglycerides with ApoB.

**Core**. The association of core protein with lipid droplets and core-RNA multimerization is among the first steps in viral assembly (Figure 2: 4 and 6). The core can oligomerize, and truncated versions have been shown to self-assemble [102], with the core-core interaction being mapped to the N-terminal part of the protein. Core oligomerization and interaction with HCV genomic RNA is thought to be an early step of virus assembly. Viral genomes containing mutations that abolish core dimerization are unable to produce infectious viruses in spite of normal replication rates [47]. A recent screen of two different compound libraries identified several hits of compounds that were able to abolish core dimerization *in vitro* [103]. Nevertheless, before these results can translate into real antiviral drugs, extensive further testing is first necessary to gauge their toxicity in cell culture and animals, and their effect on HCVcc infection.

**p7**. p7 is the smallest protein encoded by the viral genome and forms an ion-channel [56–58]. For a long time, the protein was only considered to be a non-structural protein. p7 is essential for an early step of assembly before the accumulation of intracellular infectious particles. The loss of p7 completely abolishes the production of infectious particles, and the loss of channel activity interferes with virus production in cell culture [51,56,61] (Figure 2: 3). However, a recent report suggests the additional presence of p7 in the virion, due to the fact that cation-channel inhibitors can reduce viral entry, as well as inhibit assembly. However, this effect seems rather genotype dependent [104]. One of the first drugs directed against p7 could be BIT225 (N-[5-(1-Methyl-1*H*-pyrazol-4-yl)-napthalene-2carbonyl]-guanidine), which has clearly been shown to inhibit both p7 of HCV and the related p7 of BVDV. BIT225 has antiviral activity against BVDV and has gone through different phase I clinical trials, in which it induced a modest drop in the viral titers of treated patients [105]. Moreover, it was shown that *N*-nonyl deoxygalactonojirimycin (NN-DGJ), a galactose analog coupled to a long alkyl chain that does not inhibit α-glucosidases, interferes with p7 ion channel activity and inhibits the subsequent release of infectious particles from transfected Huh-7 cells [57,106,107]. Finally, the structural data recently obtained on p7 might enable the design of new, more specific cation-channel blockers, thus making p7 a very attractive drug target [55].

**NS3 and NS5A**. NS3 combines protease and helicase activities [108,109]. The helicase domain of the protein has been shown to be essential for assembly [41]. Due to the fact that NS3 was one of the earliest drug targets in the STAT-C arsenal, several helicase inhibitors are currently under development. These molecules all inhibit replication and therefore do not present separate assembly inhibitors. NS5A was shown to play a crucial role in viral assembly in addition to its role in replication. For the moment, there is no report of a compound that targets the function of NS5A in viral assembly. A replicon based screening identified a potent NS5A inhibitor (BMS-790052) for viral replication [110]. Screening in the HCVcc system might identify molecules that target both NS3 and NS5A for viral assembly, but not replication, as presented in the next subsection.

**NS2**. NS2 is essential for a late stage in HCV assembly [49,51,53]. The protein might provide the link between early core-RNA complexes and the ER-membrane that contains the envelope glycoproteins (Figure 2: 2). Unfortunately, the protease activity is not necessary for the functions of NS2 in assembly [49,51], thus hindering the development of specific drugs until the exact requirements in NS2 for assembly have been characterized.

**Envelope glycoproteins**. The envelope proteins E1 and E2 are highly glycosylated proteins. They are incorporated into the virion in a late assembly step. Their correct folding and conformation are important for the entry of HCV, both when they interact with the different receptors, and during the fusion process (Figure 2: 5). Both viral and endogenous glycoproteins use the same cellular folding machinery to reach their native conformation. Targeting the process of glycoprotein folding represents a novel antiviral strategy. The risk of emergence of viral escape mutants would be minimal since the targets are cellular enzymes – ER α-glucosidases. ER α-glucosidases I and II process glycans that enable the nascent glycoproteins to interact with ER lectin chaperones calnexin and calreticulin, which guide the glycoprotein towards its native conformation [111,112]. Iminosugars represent a class of glucose analogs that were shown to be potent inhibitors of ER α-glucosidases and to have antiviral activity against various enveloped viruses by their interference with the folding/oligomerization process of the envelope glycoproteins [107,113–115]. *In vitro* experiments have shown an antiviral effect of different long alkyl chain imino sugar derivates [57,106,116]. Some compounds inhibit both p7 (by blocking the cation-channel activity) and the ER α-glucosidases, and probably work by a combined effect of the p7 block and their effect on E1 and E2. This occurs through the double impairment of both the assembly of infectious particles and the entry of newly generated viral particles. Two α-glucosidase-inhibitors, UT-231B (an imminosugar) and celgosivir (MX-3253 – a castanospermine prodrug), made it to phase II clinical trials, initially with very encouraging effects, especially for celgosivir. However, both have since been stopped: the phase II clinical trials with UT-231B were stopped due to low efficacy [117,118], and the clincal trials for celgosivir were stopped more recently with no precise reason given (as stated in the Migenix financial report for the first and second quarter of 2010).

### 3.2. Possible cellular targets

**ApoB, ApoE, and MTP**. It has been shown that HCV assembly is closely linked to VLDL assembly; the VLDL assembly pathway might therefore contain possible drug targets to inhibit HCV assembly. For instance, the grapefruit flavonoid narigenin has been shown to inhibit VLDL secretion both *in vitro* and *in vivo*. Additionally, narigenin can inhibit HCV secretion in cell culture. Narigenin inhibits ApoB secretion by inhibiting MTP and two enzymes involved in lipid metabolism [81]. Following this line of reasoning, anti-arteriosclerosis drugs might also be tested for their effect on HCV infection, as several of them aim to lower blood cholesterol levels by repressing cholesterol export from the liver through VLDL inhibition, e.g., by inhibiting MTP. Currently, several MTP inhibitors are in clinical trials for the treatment of hypercholesterolemia or hyperlipidemia [119,120]. Furthermore, HCV infection has tentatively been linked to higher risk for arteriosclerosis development in a Japanese cohort [121].

Nevertheless, further research into the exact relation between the HCV life cycle and lipid metabolism is necessary to evaluate potential drugs that target lipid metabolism players. Thus, it might be necessary to analyze the differences between HCV genotypes. Clinical data show increased lipid synthesis in infected liver cells and decreased VLDL secretion, as well as a higher risk for steatosis, in patients chronically infected with genotype 3, but not in patients infected with genotype 1 in [122]. This suggests that the simple inhibition of VLDL assembly might not be sufficient to successfully treat HCV infection of all genotypes, and might even exacerbate the risk of steatosis in patients infected with genotype 3.

### 3.3. Approved drugs that might potentially inhibit HCV assembly

The HCVcc system has allowed the optimization of drug screening assays, which might identify novel molecules acting at different steps in the viral lifecycle [123,124]. A recent screen of approved drugs by Gastaminza *et al*. [123] revealed the anti-assembly efficacy of two (potential) drugs from the National Institutes of Health clinical collection: pterostilbene and torimefene. Both pterostilbene and toremifene blocked viral spread in persistently infected cell cultures, but did not significantly influence entry or replication. The ratio between intracellular and extracellular infectivity indicates that the two drugs might act downstream of replication, inhibiting assembly or release. Pterostilbene is a component of blueberries corresponding to a methylated form of resveratrol and has been described to have antioxidative effects. Toremifene is a derivative of tamoxifene. Both are anti-cancer drugs. Unfortunately, as a side effect, toremifene can induce hepatitis in cancer patients, indicating a certain liver toxicity for this substance, which makes it unsuitable as an anti-HCV drug. Nevertheless, these drugs can serve as a basis for further development of novel drugs. In a second screen, Chockalingam *et. al.* reported that quinidine affected viral production by 450-fold relative to replication [124], indicating a block of assembly/release. These new drug screening assays have the potential to identify new assembly inhibitors, which either could be developed further or could shed light on the assembly process itself.

## 4. Conclusions

Even though the development of HCV specific drugs is currently an ever increasing field, these drugs are mainly directed against the targets that have already been under investigation for a long time in the replicon or HCVpp systems. Research into assembly only became possible with the development of the HCVcc system and many aspects still remain unknown. However, viral assembly presents an attractive drug target and could ensure, in combination with the current immune stimulatory treatment and protease or polymerase inhibitors, a complete clearance of the virus, or at least a significant reduction of viral titers. Although there are very few drugs already in the stages of clinical investigation, several targets have already begun to emerge. Among the viral proteins, p7 seems to be the most promising target. The increasing knowledge of the HCV-VLDL interaction has opened a new field of possible target proteins. Thus, the prevention of VLDL assembly or the inhibition of the proteins shown to play a role in HCV release might prove to be a very effective way of treating HCV. Targeting essential cellular proteins instead of viral proteins has the additional advantage of a slower emergence for resistance. This advantage is sometimes bought dearly by more severe side effects.

Since the development of the HCV cell culture system our knowledge has steadily increased. Thus, in the decade to come, we might see the same rise in antivirals targeting assembly functions, as we are currently seeing in antivirals targeting replication and polyprotein processing.
